# Assessing the Impact of Polyethylene Nano/Microplastic Exposure on Human Vaginal Keratinocytes

**DOI:** 10.3390/ijms241411379

**Published:** 2023-07-12

**Authors:** Paola Pontecorvi, Simona Ceccarelli, Fabrizio Cece, Simona Camero, Lavinia Vittoria Lotti, Elena Niccolai, Giulia Nannini, Giulia Gerini, Eleni Anastasiadou, Elena Sofia Scialis, Enrico Romano, Mary Anna Venneri, Amedeo Amedei, Antonio Angeloni, Francesca Megiorni, Cinzia Marchese

**Affiliations:** 1 Department of Experimental Medicine, Sapienza University of Rome, Viale Regina Elena 324, 00161 Rome, Italy; paola.pontecorvi@uniroma1.it (P.P.); simona.ceccarelli@uniroma1.it (S.C.); cece.fabrizio@gmail.com (F.C.); simona.camero@uniroma1.it (S.C.); laviniavittoria.lotti@uniroma1.it (L.V.L.); giulia.gerini@uniroma1.it (G.G.); maryanna.venneri@uniroma1.it (M.A.V.); antonio.angeloni@uniroma1.it (A.A.); francesca.megiorni@uniroma1.it (F.M.); 2 Department of Experimental and Clinical Medicine, University of Florence, Largo Brambilla 3, 50134 Florence, Italy; elena.niccolai@unifi.it (E.N.); giulia.nannini@unifi.it (G.N.); amedeo.amedei@unifi.it (A.A.); 3 Department of Clinical and Molecular Medicine, Sapienza University of Rome, Via di Grottarossa 1035, 00189 Rome, Italy; eleni.anastasiadou@uniroma1.it; 4 Department of Innovative Technologies in Medicine and Dentistry, University “G. D’Annunzio” Chieti—Pescara, Via dei Vestini 31, 66100 Chieti, Italy; elenasofia.scialis@gmail.com; 5 Department of Sense Organs, Sapienza University of Rome, Viale del Policlinico 155, 00161 Rome, Italy; enrico.romano@uniroma1.it

**Keywords:** polyethylene, nanoplastics, microplastics, period products, vaginal keratinocytes, nano/microparticles uptake

## Abstract

The global rise of single-use throw-away plastic products has elicited a massive increase in the nano/microplastics (N/MPLs) exposure burden in humans. Recently, it has been demonstrated that disposable period products may release N/MPLs with usage, which represents a potential threat to women’s health which has not been scientifically addressed yet. By using polyethyl ene (PE) particles (200 nm to 9 μm), we showed that acute exposure to a high concentration of N/MPLs induced cell toxicity in vaginal keratinocytes after effective cellular uptake, as viability and apoptosis data suggest, along with transmission electron microscopy (TEM) observations. The internalised N/MPLs altered the expression of junctional and adherence proteins and the organisation of the actin cortex, influencing the level of genes involved in oxidative stress signalling pathways and that of miRNAs related to epithelial barrier function. When the exposure to PE N/MPLs was discontinued or became chronic, cells were able to recover from the negative effects on viability and differentiation/proliferation gene expression in a few days. However, in all cases, PE N/MPL exposure prompted a sustained alteration of DNA methyltransferase and DNA demethylase expression, which might impact epigenetic regulation processes, leading to accelerated cell ageing and inflammation, or the occurrence of malignant transformation.

## 1. Introduction

Plastic has been one of the most revolutionary inventions of the last century, and its employment has had a tremendous impact on human everyday life [1]. It is inexpensive, strong, durable, lightweight, and resistant to degradation [2]. Due to these properties, along with waste mismanagement, plastic pollution has become an urgent problem nowadays [1]. Plastic-containing products, when exposed to biological, chemical and physical conditions during and after usage, can break up into pieces smaller than 1 mm, the microplastics (MPLs), or smaller than 1000 nm, the nanoplastics (NPLs) [3].

Some of the most used industrial plastics are polyethene, also known as polyeth-ylene (PE), polypropylene (PP), polystyrene (PS), polyvinyl chloride (PVC), polyethene terephthalate (PET), and polymethyl methacrylate (PMMA), known as acrylic [4]. As N/MPLs include contaminants with diverse physicochemical properties, making certain predictions on the potential adverse effects of N/MPLs on health is a hard challenge. In the last 3 years, a growing number of studies have highlighted the correlation between N/MPLs and different pathological processes. The toxic effects of N/MPLs on marine organisms, including invertebrates, have been widely studied [5,6]. However, there is a paucity of information regarding the human health implications of exposure to N/MPLs. The main N/MPL intake occurs through the oral and nasal cavities. For humans, the ingestion of airborne N/MPLs represents the predominant source of exposure compared to the consumption of food containing N/MPLs; it is estimated that up to 70.000 airborne particles are ingested by an individual in a year [7]. N/MPL ingestion, revealed by their detection in dietary products and drinking water, has been associated with different issues in the intestine, such as direct physical damage, increased gut permeability, alteration of the microbiota composition and local inflammatory response. N/MPLs, because of their high surface area, could harbour or absorb many different types of chemicals and pathogens from the environment. Moreover, the reactivity and toxicity of particles increase along with the surface area to mass ratio, so interactions with biological barriers become more likely [8]. As for skin absorption, certain cosmetic products, such as toothpaste, and face and body scrubs, contain primary N/MPLs used as exfoliators for the skin and teeth, the majority of them made of PE [9]. The glitters used in make-up are usually PET-containing plastics [10]. Indeed, the continuous use of cosmetics with incorporated N/MPL particles over a prolonged time may result in premature ageing of skin cells. Other personal care products used by a large part of society are period products. Recently, the fragmentation and release of N/MPLs from period products has been demonstrated [11], by identifying synthetic polymers in 7 out of 12 products tested (polyester, PP, PE and nylon). Considering the median number of particles found in the tested products, it can be estimated that a woman using tampons could be exposed to 86 trillion fragmented synthetic polymers over a lifetime of product use. Apart from their contribution to environmental pollution with N/MPLs, it has not been shown whether plastic-containing period products releasing N/MPLs during usage may interact with the respective tissues [12]. This issue is of concern because such amounts of N/MPLs have the potential to be released inside the vagina and, therefore, to trigger local chronic effects. In addition, arteries, blood vessels and lymphatic vessels are abundant in the vaginal wall, which allows for the direct transfer of chemicals to the blood through peripheral circulation [13]. Indeed, Heather et al. recently demonstrated that plastic particles are bioavailable for uptake into the human bloodstream [14]. Finally, the identification of N/MPLs in the human placenta has been significant [15], since it could negatively affect foetal development [16]. Taken together, these studies suggested that tampons and pads could be a non-neglectable source of N/MPLs found in several female body districts.

The aim of the present study is to assess the possible adverse effects exerted on human vaginal mucosa by ordinary contact with products containing N/MPLs. By employing the VK2 E6/E7 vaginal cell line, we tested different exposure conditions to PE N/MPLs, in order to mimic common usage habits of period products. We performed cell viability assays and cytofluorimetric analyses to assess cell death processes; we studied cell morphology alterations through optical and fluorescence microscopy. Moreover, we assessed N/MPL uptake by vaginal cells by transmission electron microscopy (TEM). In order to identify changes in the expression of genes related to cell proliferation, differentiation, senescence and apoptosis, as well as epigenetic regulation, we performed quantitative real-time PCR (RT-qPCR) analyses and Western blot assays. Additionally, the inflammatory response was evaluated in cell culture supernatants through multiplex cytokine analysis. Finally, we performed a colony formation assay to study the occurrence of cell transformation in VK2 E6/E7 chronically exposed to PE N/MPLs. Our results suggest that, apart from being potentially pollutant for the environment, the use of disposable period products may be dangerous for human health; hence, efforts should be made towards the substitution of plastic bulk material, preferring natural fibres such as cotton and cellulose.

## 2. Results

### 2.1. Acute Exposure to PE N/MPLs Affects Vaginal Keratinocyte Morphology and Vitality

**Figure 1:**
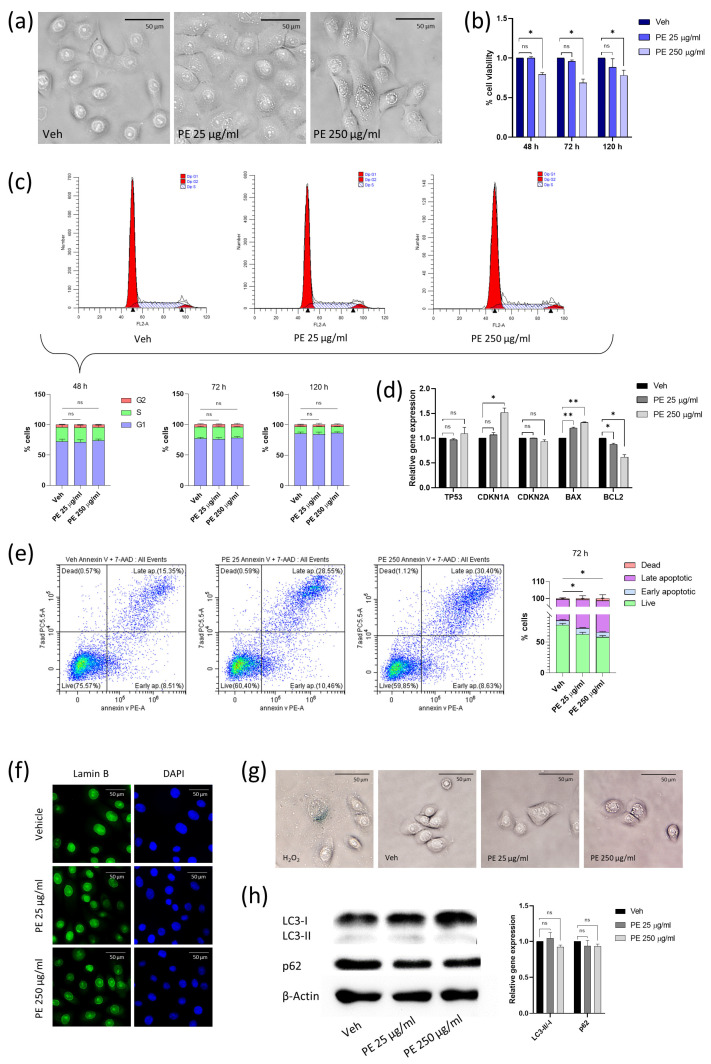
Evaluation of vaginal keratinocyte morphology and viability after acute PE N/MPL exposure. (**a**) Representative images of VK2 E6/E7 treated for 48 h with Tween20 (Veh; 0.0025%) or PE N/MPLs at low and high concentrations (25 and 250 μg/mL). Pictures were taken at 40× magnification. Scale bars are 50 μm. (**b**) Graphical representation of the percentage of viable vaginal keratinocytes after 48, 72, and 120 h vehicle or PE N/MPL exposure obtained with a Trypan blue dye exclusion assay. (**c**) Representative plots of FACS cell cycle analysis at 48 h and percentage histograms at 48, 72, and 120 h exposure to vehicle or PE N/MPLs. Arrowheads in FACS plots indicate the ideal position of G1 and G2 peaks. (**d**) Histograms showing gene expression levels of cell cycle and apoptosis markers in cells treated for 48 h with PE N/MPLs compared to vehicle. (**e**) Representative dot plots and histograms displaying the percentage of apoptotic cells upon vehicle and PE particle exposure for 72 h. Blue corresponds to areas of lower cell density, red and orange are areas of high cell density, green and yellow indicate mid-range density areas. (**f**) Illustrative IF pictures for Lamin B (green, left) and DAPI (blue, right) staining of VK2 E6/E7 treated with vehicle or PE N/MPLs 25–250 μg/mL for 72 h. Pictures were taken at 40× magnification. Scale bars are 50 μm. (**g**) Representative pictures of β-galactosidase staining of VK2 E6/E7 exposed to vehicle or PE 25 and 250 μg/mL for 72 h. H_2_O_2_ treatment was used as a positive control. Pictures were taken at 40× magnification. Scale bars are 50 μm. (**h**) Illustrative Western blot and densitometric analysis for the autophagy markers LC3 B and p62 in VK2 E6/E7 exposed to vehicle or PE 25 and 250 μg/mL for 48 h (β-Actin was used as loading control) (Figure S2). For all the experiments, statistical analysis was conducted by unpaired, two-tailed Student’s *t*-test (*n* > 3) with “ns” non-significant, * *p* ≤ 0.05 and ** *p* ≤ 0.005.

In order to facilitate studies on PE N/MPL biological effects, we selected two different particle concentrations (25 and 250 μg/mL) based on the findings of Gopinath et al. [9] and Schmidt et al. [17] on human and mouse skin keratinocytes. After 48 h of treatment, we noted a morphological change in VK2 E6/E7 cells, which showed peculiar vesicular formations (Figure 1a). For PE 25 μg/mL, vesicles were rare and heavenly distributed in the cytoplasm, while for PE 250 μg/mL, their number notably increased, and vesicles accumulated around the nucleus. We investigated the potential effect on cell viability of PE N/MPLs, by exposing VK2 E6/E7 for 48, 72, and 120 h to the two-particle concentrations. Notably, we observed a significant decrease in the percentage of viable cells at all three timepoints for the highest PE N/MPL concentration with respect to vehicle controls, with a peak at 72 h (Figure 1b). Hence, we performed a cell cycle analysis to test if the diminished cell viability may be related to a reduction in the number of actively proliferating cells. After 48, 72, and 120 h of treatment, we did not highlight significant alterations of cell cycle phases (Figure 1c) in treated cells with respect to controls. The percentage of VK2 E6/E7 in the G1 phase increased overtime because cells gradually reached the confluence through the three timepoints. The early dysregulation of cell cycle and apoptosis markers expression was assessed after a 48 h PE N/MPL exposure through RT-qPCR. We observed a significant upregulation of Cyclin-dependent kinase inhibitor 1A (*CDKN1A*, encoding for p21) mRNA levels for PE 250 μg/mL treatment compared to vehicle, a substantial gradual upregulation of Bcl-2-associated X protein (*BAX*) levels with the increase of PE N/MPL concentration, and a matching statistically significant B-cell lymphoma 2 (*BCL2*) downregulation with respect to controls (Figure 1d). Finally, tumour protein 53 (*TP53*, encoding for p53) and Cyclin-dependent kinase inhibitor 2A (*CDKN2A*, encoding for p16) mRNA levels were not significantly affected at both PE particle concentrations with respect to control.

*BAX* and *BCL2* encode for two cytoplasmatic proteins, which act as a promoter and an inhibitor of apoptosis, respectively. Hence, we performed an Annexin V staining analysis to confirm the occurrence of apoptotic cell death after 72 h exposure and highlighted that the percentage of live cells substantially decreased in PE N/MPL-treated cells with respect to vehicle controls for both low and high particle concentrations, while the percentage of late apoptotic vaginal keratinocytes significantly increased with PE 25 and 250 μg/mL treatment (Figure 1e), confirming the results obtained with Trypan blue dye exclusion assays. To exclude the occurrence of cell senescence following PE N/MPL exposure, we performed β-galactosidase and Lamin B staining on VK2 E6/E7 treated with vehicle, 25, or 250 μg/mL of PE particles for 72 h. Indeed, we neither detected blue precipitate formation, nor Lamin B fluorescent signal changes in N/MPL exposed cells with respect to control (Figure 1f,g). In conclusion, to clarify the nature of the perinuclear vesicular formations, we assessed the expression of two validated autophagy markers through Western blot analysis at 48 h from treatment (Figure 1h). The immunoblot did not highlight any significant alteration of Microtubule-associated protein 1A/1B light chain 3 (LC3) II/I ratio and Ubiquitin-binding protein 62 (p62) expression, as shown by the corresponding densitometric analysis.

### 2.2. Cellular Uptake of N/MPLs during Acute Exposure to PE Particles Is Directly Proportional to PE N/MPL Concentration and Protein Corona Formation

**Figure 2:**
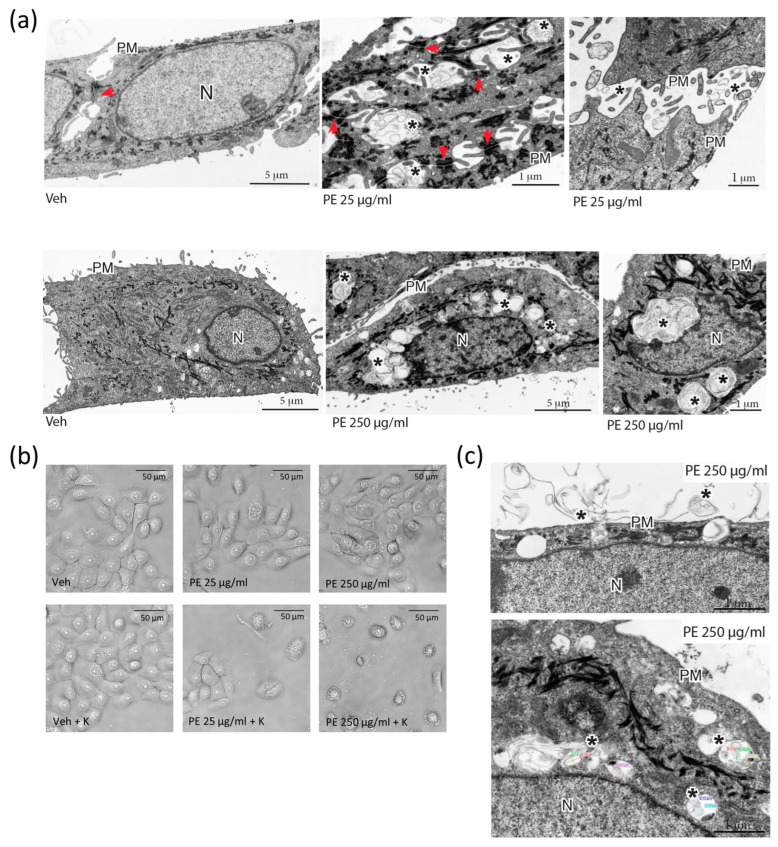
Assessment of cellular uptake of PE N/MPLs by vaginal keratinocytes upon acute exposure. (**a**) TEM analysis of VK2 E6/E7 cells after 72 h of treatment with 25 and 250 μg/mL of PE N/MPLs or vehicle. Upper panel: From left to right, VK2 E6/E7 cells exposed to vehicle versus 25 μg/mL of PE N/MPLs. In the latter, extracellular accumulation of N/MPLs (asterisks) is evident between cells, in proximity to surface microvilli and intercellular junctions (red arrows). Lower panel: From left to right, VK2 E6/E7 cells exposed to vehicle versus 250 μg/mL of PE N/MPLs. In the latter, the N/MPLs (asterisks) are principally localised within cytoplasmic endocytic structures, often distributed around the nucleus. Two different vehicle pictures are present in the panel, since we performed the experiment with two distinct vehicle volumes matching the two PE N/MPLs concentrations. (**b**) Optical microscopy analysis of VK2 E6/E7 cells exposed for 72 h to vehicle versus PE N/MPLs coated with 0.2% human keratin in aqueous solution. (**c**) Ultrastructural analysis of the uptake of PE N/MPLs (250 μg/mL) coated with 0.2% human keratin in aqueous solution. TEM micrographs show PE N/MPLs (asterisks) deposited on the plasma membrane of VK2 E6/E7 cell, with initial uptake in forming vacuoles (**top**), and internalisation within peripheral and perinuclear endosome-like organelles (**bottom**). The NP mean diameter, reported with coloured lines in the figure (**bottom**), is consistent with that of the N/MPLs used in the experiment. PM: plasma membrane; N: nucleus. Scale bar: 1 μm.

TEM was employed to deepen insight into the nature of PE N/MPL treatment-induced perinuclear vesicle aggregates. VK2 E6/E7 cells were treated for 72 h with the two concentrations of PE N/MPLs or vehicle control, and then 80% confluent cells were fixed and stained for image acquisition. At the lowest particle concentration, PE N/MPLs aggregated on the surface of cell membranes and accumulated between cells, near the adherens junctions, but did not enter the cytoplasm (Figure 2a, upper panel, adherens junctions indicated with arrowheads, PE aggregates marked with asterisks). Remarkably, we observed an apparent increase in cytokeratin expression in PE 25 μg/mL-treated cells (electron-dense filaments). In addition, at the highest particle concentration, PE N/MPL intracellular aggregates were clearly visible in endosome-resembling structures gathered around the nucleus (Figure 2a, lower panel, asterisks).

Biological macromolecules, especially proteins, tend to adsorb on N/MPL surfaces at a rate that is directly proportional to the availability of biomolecules, and the consequent development of a protein corona may foster the uptake of the particles and amplify their cytotoxic effects on cells [9]. In order to assess the impact of protein corona formation on PE N/MPL cellular uptake, we coated the particles with 0.2% human keratin aqueous solution for 24 h at 4 °C before using them for 72 h treatments. Firstly, we observed a huge impact on cell morphology and viability of treated VK2 E6/E7 with optical microscopy, by identifying an increasing number of apoptotic-like cells, which was positively related to PE N/MPL concentration (Figure 2b). Furthermore, TEM acquisitions highlighted spherical material surrounded by amorphous filaments on the outside of the cell, which are likely to be PE N/MPLs covered with proteins (Figure 2c, upper panel, asterisks). Notably, the mean size of PE particles trapped into the endosomal-like structures was 180 nm (Supplementary Materials, Figure S1 and Table S1), confirming NP cellular uptake at 72 h, which in turn may be responsible for the cell toxicity peak observed at 250 μg/mL (Figure 2c, lower panel, asterisks). As a result of protein corona formation on the N/MPLs, the cells might have recognised them as protein aggregates, hence activating the engulfment process.

### 2.3. PE N/MPL Acute Exposure Impacts on Vaginal Keratinocyte Cytoskeleton Structure, Redox Homeostasis, and Cell Stress

**Figure 3:**
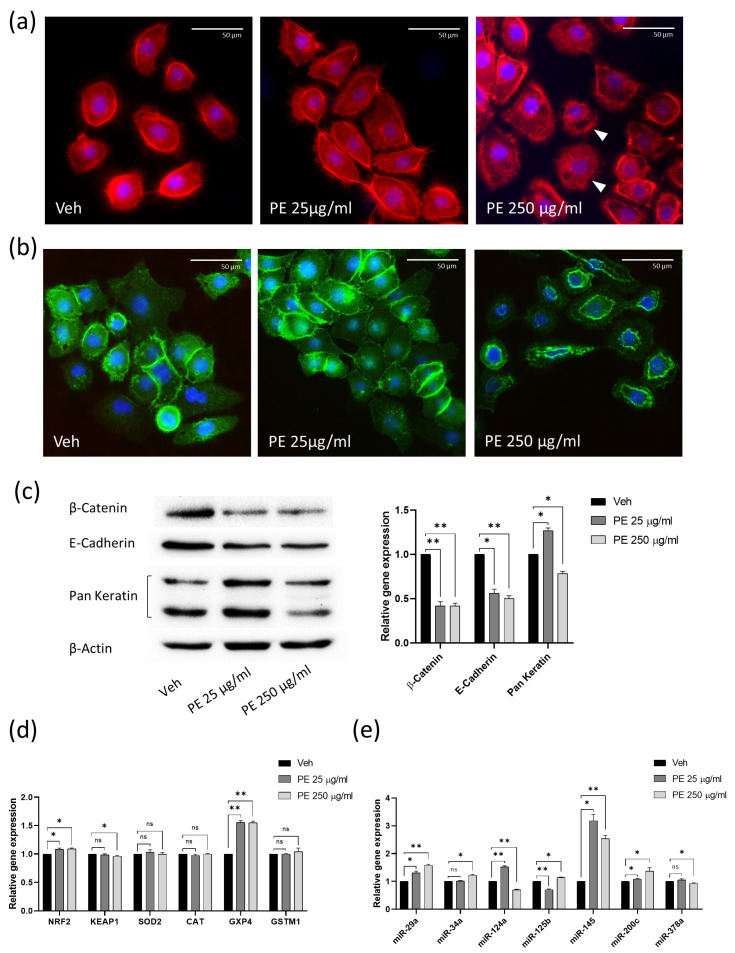
Analysis of the effects of PE N/MPL acute exposure on VK2 E6/E7 cytoskeleton and cell stress pathways. (**a**) Representative IF acquisitions showing actin cytoskeleton of vaginal keratinocytes treated with vehicle or PE N/MPLs at 25–250 μg/mL for 72 h. Actin is stained in red with DyLight™ 554 Phalloidin, and nuclei are stained in blue with DAPI. Arrowheads indicate gaps in the cytoskeletal net. Pictures were taken at 40× magnification. Scale bars are 50 μm. (**b**) Illustrative IF pictures showing β-Catenin localisation in VK2 E6/E7 exposed to vehicle or PE N/MPLs 25–250 μg/mL for 72 h. β-Catenin is stained in green with Alexa Fluor™ 488, while nuclei are stained in blue with DAPI. Acquisitions were obtained at 40× magnification. Scale bars are 50 μm. (**c**) Representative Western blot and corresponding densitometry for β-Catenin, E-cadherin, and Pan Keratin expression in vaginal keratinocytes exposed to vehicle or PE N/MPLs at 25–250 μg/mL for 72 h. β-Actin was used as a loading control (Figure S2). (**d**) RT-qPCR graphs showing relative expression of stress and redox homeostasis genes in VK2 E6/E7 exposed for 48 h to vehicle or PE 25–250 μg/mL. GAPDH mRNA level was used as endogenous control. (**e**) Histograms displaying epithelial barrier function-related microRNA expression in vaginal keratinocytes treated with vehicle or low and high PE particle concentrations for 48 h. U6 snRNA levels were employed as endogenous control. All the statistical analyses were conducted by unpaired, two-tailed Student’s *t*-test (*n* = 3) with “ns” non-significant, * *p* ≤ 0.05 and ** *p* ≤ 0.005.

The considerable modification in cell morphology following PE N/MPL uptake may be a cause and a consequence of cytoskeleton alteration. In order to test this hypothesis, we investigated the actin cytoskeleton through phalloidin immunofluorescence staining on VK2 E6/E7 cells treated with PE particles for 72 h. Acquisitions highlighted well-defined actin cortex, retraction fibres, lamellipodia and filipodia in vehicle-treated vaginal keratinocytes (Figure 3a). As for PE 25 μg/mL exposed cells, we did not observe significant changes in actin structures compared to control, whilst for cells exposed to the highest concentration of PE particles, a transient breakdown of the actin cytoskeleton was identified, as suggested by the rarefaction of actin cortex, the absence of long actin stress fibres, and more diffuse intracellular staining (Figure 3a, arrowheads). The actin cytoskeleton regulates the creation and stabilisation of β-catenin/E-cadherin complexes between adherent cells, which play a fundamental role in maintaining epithelial integrity and in controlling the activation of the wingless (Wnt)/β-catenin pathway, in which cytoplasmic β-catenin translocates into the nucleus and functions as an activator for transcription factors involved in cellular adhesion, tissue morphogenesis, and tumour development [18]. By using immunofluorescence staining, we highlighted a well-dispersed β-catenin signal in vehicle-exposed cells, evenly distributed between the inner layer of the membrane, the cytoplasm, and the nucleus (Figure 3b). After the exposure to PE 25 μg/mL, we observed the increment of β-catenin membranous signal at cell–cell contacts with respect to control. Interestingly, we documented an increase in perinuclear accumulation for β-catenin staining in PE 250 μg/mL-treated vaginal keratinocytes with respect to cells exposed to vehicle and PE 25 μg/mL. In addition, we assessed the expression levels of selected cytoskeleton-associated proteins through Western blot analysis at 72 h of treatment. Significant downregulation of β-catenin and E-cadherin protein levels was registered after acute PE N/MPL exposure for both PE particle concentrations compared to control (Figure 3c). Cytokeratins are heteropolymeric structural proteins which form the majority of intermediate filaments in keratinocytes, which protect cells from mechanical stress [19]. We assessed the total protein level of these molecules by using a Pan Keratin antibody, and we found a significant upregulation of cytokeratins in VK2 E6/E7 exposed to the lowest concentration of PE particles with respect to control, confirming the findings obtained by TEM analysis. On the contrary, the levels of cytokeratins were significantly downregulated in cells treated with PE 250 μg/mL with respect to vehicle-exposed cells, as shown by the densitometric analysis (Figure 3c).

Considering the substantial impact of PE N/MPL exposure on cell morphology and vitality, we looked for the occurrence of gene expression alteration of different cell stress markers. We performed RT-qPCR analysis on vaginal keratinocytes treated with PE N/MPLs for 48 h for Nuclear factor erythroid 2-related factor 2 (*NRF2*), a regulator of cellular resistance to oxidants, and Kelch-like erythroid cell-derived protein with CNC homology-associated protein 1 (*KEAP1*), which negatively regulates NRF2 by ubiquitination–proteasomal degradation. NRF2 controls the expression of antioxidant response element (ARE)-dependent genes to counteract reactive oxygen and nitrogen species deriving from internal metabolism and environmental toxicant exposure [20]. We then assessed the mRNA levels for Superoxide dismutase 2 (*SOD2*), Catalase (*CAT*), Glutathione peroxidase 4 (*GPX4*) and Glutathione S-transferase mu 1 (*GSTM1*). The main role of SOD2 is to convert the superoxide by-products of oxidative phosphorylation into oxygen and hydrogen peroxide (H_2_O_2_), which is then catalysed into H_2_O by CAT and GPX4, a process supported by enzymes such as GSTM1. We highlighted a significant upregulation of *NRF2* for both PE N/MPL concentrations with respect to control and a statistically significant downregulation of *KEAP1* at the highest PE particle concentration compared to vehicle (Figure 3d). With regards to the *SOD2*, *CAT* and *GSTM1* mRNA levels, we did not observe any considerable fluctuation in N/MPL-treated vaginal keratinocytes compared to control. Remarkably, RT-qPCR analysis revealed a substantial upregulation of *GPX4* expression in cells exposed to both low and high concentrations of PE N/MPLs (Figure 3d). GPX4 is an enzyme that protects cells against membrane lipid peroxidation, and it has a less restricted dependence on glutathione as a reducing substrate compared to other glutathione peroxidases [21]. Considering microRNAs (miRNAs) could be early markers of biological process dysregulation, we analysed an array of miRNAs related to epithelial barrier function, cell stress and inflammation in keratinocytes through RT-qPCR analysis on VK2 E6/E7 cells treated with PE N/MPLs for 48 h. Specifically, miR-34a has been previously associated with physiological skin ageing [22], whilst it has been shown that the miR-29 family exerts a range of regulatory functions by controlling apoptosis, proliferation, and differentiation processes [23]. We highlighted a significant upregulation of miR-29a for both PE N/MPL concentrations and an increase in miR-34a levels in cells exposed to PE 250 μg/mL compared to control (Figure 3e). It has been demonstrated that miR-124a is implicated in wound healing and skin ageing [24], that miR-125b plays a role in keratinocyte differentiation and proliferation [22], and that miR-145 regulates cell proliferation and secretion of chemokines in normal human epidermal keratinocytes [25]. Interestingly, we noted a significant dysregulation of miR-124a and miR-125b levels compared to control, with an opposite trend for PE 25 μg/mL- and PE 250 μg/mL- treated cells, whilst a considerable upregulation of miR-145 was observed for both low and high concentration PE N/MPL-exposed cells (Figure 3e). Magenta et al. showed that miR-200c is upregulated by reactive oxygen species (ROS) and is responsible for apoptosis and senescence in skin cells [26], while Xia P. et al. proved that miR-378a is implicated in Interleukin 17A (IL-17A)-driven skin inflammation [27]. Our analyses highlighted a significant gradual upregulation of miR-200c, which increases with particle concentration, in PE N/MPL-treated cells with respect to vehicle, and a slight alteration of miR-378a levels in PE 250 μg/mL exposed vaginal keratinocytes compared to control (Figure 3e).

### 2.4. Vaginal Keratinocytes Could Recover from the Effects of Discontinuous PE N/MPL Exposure, Which Leaves a Mark on Epigenetic Enzymes Expression Profile

**Figure 4:**
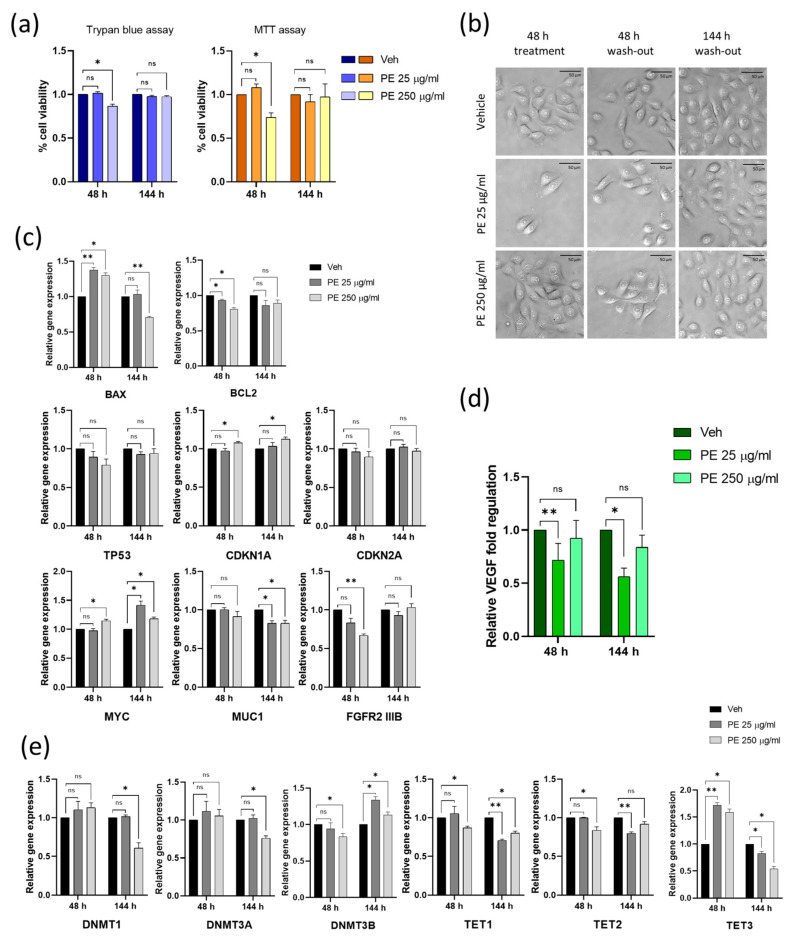
Assessing the impact of discontinuous PE N/MPL exposure on cell viability, morphology, proliferation, inflammation, and epigenetic regulation in vaginal keratinocytes. (**a**) Graphs showing the percentage of viable cells upon 48 h of exposure to low and high concentrations of PE particles and 144 h post-wash-out obtained via Trypan blue and MTT assays. (**b**) Representative pictures of VK2 E6/E7 exposed to vehicle or PE N/MPLs for 48 h and 144 h after wash-out taken throughout the experimental points at 40× magnification. Scale bars are 50 μm. (**c**) Histograms for RT-qPCR analysis displaying a relative expression of apoptosis and cell cycle markers, and proliferation/differentiation-associated genes in VK2 E6/E7 exposed to vehicle or PE 25–250 μg/mL for 48 h and after 144 h wash-out. (**d**) Graphs showing relative fold regulation for VEGF concentrations detected in cell culture supernatants at 48 h of exposure to PE 25–250 μg/mL or vehicle and at 144 h from wash-out. (**e**) Relative gene expression of epigenetic regulation enzymes in vaginal keratinocytes exposed to PE 25–250 μg/mL or vehicle for 48 h and at 144 h post-wash-out. All the statistical analyses were conducted by unpaired, two-tailed Student’s *t*-test (*n* ≥ 3) with “ns” non-significant, * *p* ≤ 0.05 and ** *p* ≤ 0.005.

In order to assess the resilience of VK2 E6/E7 after acute PE N/MPL exposure, we treated them for 48 h with PE particles and then washed out the N/MPLs by culturing the cells for 144 h in an N/MPL-free medium. Firstly, cell vitality and morphology were investigated through Trypan blue dye exclusion assay and optical microscopy. At 48 h from treatment, a significant decrease in cell viability was observed for PE 250 μg/mL-treated cells with respect to control, whilst at 144 h of wash-out, no differences were highlighted for both concentrations compared to vehicle-treated cells. These findings were validated by MTT assay (Figure 4a). With regards to cell morphology, we observed the formation of perinuclear vesicles after a 48 h treatment in PE 250 μg/mL exposed cells, which were still present at 48 h after wash-out, while they completely disappeared at 144 h from a wash-out, where VK2 E6/E7 regained a normal cell phenotype, comparable to that of vehicle-treated cells (Figure 4b). Gene expression of apoptosis and cell cycle markers was assessed 48 h post-PE N/MPL treatment and at 144 h from wash-out through RT-qPCR (Figure 4c). At 48 h, *BAX* mRNA levels were significantly upregulated, whilst *BCL2* expression significantly decreased for both PE particle concentrations with respect to control, as demonstrated in Section 2.1. At 144 h, *BAX* was significantly downregulated in PE 250 μg/mL-treated cells with respect to control, while *BCL2* expression was restored at levels comparable to vehicle-exposed cells. As for cell cycle markers, *TP53* and *CDKN2A* were not influenced by PE N/MPL exposure at 48 h or at 144 h. However, *CDKN1A* mRNA levels were significantly upregulated at 48 h from treatment (as demonstrated in Section 2.1), and they remained so at 144 h from wash-out in PE 250 μg/mL exposed cells with respect to control.

Moreover, we assessed the expression levels of genes related to keratinocyte proliferation and differentiation. Many studies have demonstrated that Myelocytomatosis oncogene product (c-Myc) plays a positive role in keratinocyte proliferation, but it can also stimulate terminal differentiation as a safety mechanism that protects keratinocytes from uncontrolled proliferation; the level and duration of Myc activation exerts different effects [28]. We observed a significant upregulation of *MYC* expression in PE 250 μg/mL-treated cells compared to control at 48h, and a sustained increase in *MYC* levels for both PE particles concentration at 144 h from wash-out with respect to vehicle. Mucin 1 (MUC1) is a transmembrane glycoprotein highly expressed in vaginal epithelial tissue, mainly involved in the creation of a protective barrier. In addition, the MUC1 cytoplasmic tail has been shown to associate with β-catenin and other signalling molecules, suggesting a potential role for MUC1 in cell signalling [29]. At 48 h exposure, we did not observe significant alterations in *MUC1* mRNA levels in VK2 E6/E7 cells treated with PE particles with respect to control, whilst at 144 h from wash-out, *MUC1* expression was significantly downregulated in cells treated with both low and high PE N/MPL concentration in comparison to vehicle. The expression of Fibroblast growth factor receptor 2, isoform 3B (*FGFR2 IIIB*), also known as keratinocyte growth factor receptor (KGFR), controls the proliferative–differentiative program in skin keratinocytes, as previously demonstrated [30,31]. Herein, RT-qPCR analysis demonstrated a significant downregulation of *FGFR2 IIIB* mRNA in VK2 E6/E7 treated with PE 250 μg/mL with respect to control at 48 h, whilst at 144 h from wash-out, *FGFR2 IIIB* expression returned at levels comparable to that of vehicle-exposed cells (Figure 4c).

To investigate the occurrence of inflammatory reactions upon PE N/MPL treatment, we quantified the levels of selected chemokines and cytokines secreted by VK2 E6/E7 in cell culture supernatants at 48 h exposure and 144 h after wash-out. We defined a specific panel, including those most involved in skin inflammation and correlated diseases. For instance, high amounts of thymic stromal lymphopoietin (TSLP) in patients with atopic dermatitis activate T helper (Th) cells to produce Th2 cytokines (e.g., Interleukine (IL)-4, IL-5), which in turn can directly lead keratinocytes to further produce more TSLP [32,33]. Keratinocytes from injured skin highly express IL-33 [34], while IL-6 has been shown to induce naive CD4+ T cells differentiation into Th17 cells [35]. Tumour necrosis factor (TNF)-α induces keratinocytes to perpetually produce chemokines such as chemokine C-X-C motif ligand 1 (CXCL1) to recruit immune cells to the damaged site, thus triggering skin inflammation [36,37]. Finally, it is well established that in many skin conditions closely related to angiogenesis or chronic inflammation, there is a prominent induction of vascular endothelial growth factor (VEGF) in epidermal keratinocytes [38,39]. Hence, we dosed the concentration of TSLP, CXCL1, CXCL10, TNF-α, VEGF, IL-1α, IL-1β, IL-6, IL-12 and IL-33. Interestingly, VEGF was the only factor showing detectable concentrations at the multiplex analysis (pg/mL), which highlighted a significant decrease of VEGF levels in cells treated for 48 h with PE 25 μg/mL with respect to control. This trend was also maintained at 144 h after wash-out (Figure 4d).

To assess the influence of PE N/MPL exposure on a broader scale, we analysed the expression of enzymes controlling the methylation status of DNA on VK2 E6/E7 cells treated with PE particles for 48 h and washed out from them for 144 h (Figure 4e). The maintenance DNA methyltransferase (DNMT) 1 and the de novo methyltransferases DNMT3A and DNMT3B are essential enzymes that catalyse the addition of methylation marks to cytosine-5 of genomic DNA. They play an important role in maintaining genome stability, and their activity is highly regulated [40]. The Ten eleven translocation (TET) enzymes oxidise 5-methylcytosines (5mCs) and promote locus-specific reversal of DNA methylation [41]. Both protein families also exert non-enzymatic functions. Indeed, DNMT and TET enzymes can interact to dynamically regulate the binding of transcription factors to DNA [40]. Aberrant expression of DNMTs and TETs may lead to the disruption of DNA methylation patterns, which are closely associated with many forms of cancer and chronic diseases [42]. RT-qPCR analysis showed that *DNMT1* and *DNMT3A* were not influenced by PE particle exposure at 48 h of treatment, whilst a significant downregulation of both was highlighted at 144 h from wash-out for PE 250 μg/mL exposed cells with respect to control. On the contrary, *DNMT3B* mRNA levels were significantly downregulated at 48 h for PE 250 μg/mL-treated cells, and this trend was reversed at 144 h from wash-out, with a considerable increase in *DNMT3B* mRNA levels for PE N/MPL-treated cells at both concentrations in comparison to control. As for *TET1* expression, a significant downregulation is observed for PE 250 μg/mL-treated cells compared to vehicle at both experimental timepoints, with a substantial decrease of *TET1* also in PE 25 μg/mL exposed cells at 144 h from wash-out. With regards to *TET2* mRNA levels, we observed a significant fluctuation for PE 250 μg/mL-treated cells at 48 h exposure, whilst a substantial downregulation was observed for PE 25 μg/mL exposed VK2 E6/E7 at 144 h wash-out with respect to vehicle. Finally, *TET3* expression considerably increased at 48 h exposure and substantially decreased at 144 h from wash-out for both low and high concentrations of PE particles-treated cells with respect to control. These findings suggest that the impact of PE N/MPLs on the expression of epigenetic enzymes is not directly proportional to particle concentration in vaginal keratinocytes.

### 2.5. Chronic Exposure to PE N/MPLs Allows Keratinocytes to Adapt to PE Particle Toxicity Overtime

**Figure 5:**
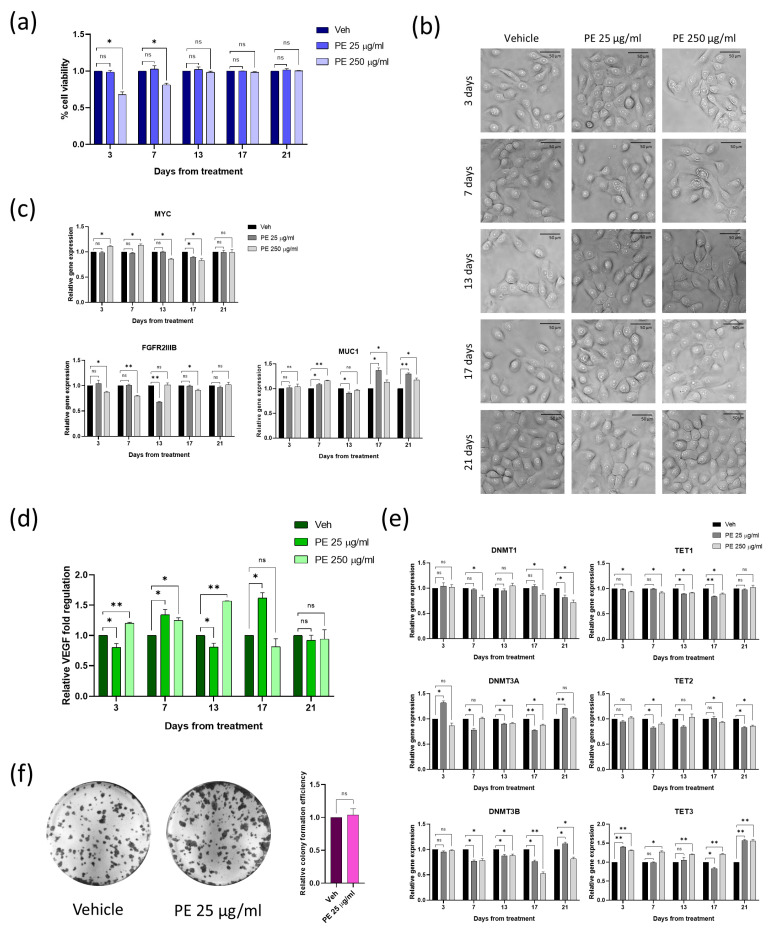
Assessment of the impact of PE N/MPL chronic exposure on vaginal keratinocytes viability, morphology, proliferation, inflammation, epigenetic regulation, and transformation potential. (**a**) Diagrams showing the results of the Trypan blue assay with the percentage of viable cells for VK2 E6/E7 chronically exposed to low and high concentrations of PE N/MPLs or vehicle for 21 days. (**b**) Illustrative images of vaginal keratinocytes treated with vehicle or PE 25–250 μg/mL at day 3 to 21 of exposure. Pictures were taken at 40× magnification. Scale bars are 50 μm. (**c**) Graphs for RT-qPCR analysis displaying a relative expression of proliferation/differentiation-associated genes in VK2 E6/E7 chronically exposed to vehicle or PE 25–250 μg/mL for 21 days. (**d**) Histograms showing relative fold regulation for VEGF concentrations detected in VK2 E6/E7 cell culture supernatants at 3 to 21 days of exposure to PE 25–250 μg/mL or vehicle. (**e**) Relative gene expression of epigenetic regulation enzymes in vaginal keratinocytes exposed to PE 25–250 μg/mL or vehicle for 21 days. (**f**) Representative images of colony formation assays of VK2 E6/E7 chronically exposed to PE 25 μg/mL or vehicle for one month and two weeks. Staining was performed with Crystal violet. Relative absorbance for solubilised Crystal violet was reported on a diagram. Statistical analyses were conducted by unpaired, two-tailed Student’s *t*-test (*n* = 3) with “ns” non-significant, * *p* ≤ 0.05 and ** *p* ≤ 0.005.

To study the effects of continuous PE N/MPL exposure, we treated VK2 E6/E7 cells with PE particles uninterruptedly for three weeks, and analysed cell viability, proliferation, and epigenetic parameters at five different timepoints. First, via Trypan blue dye exclusion assay, we noticed that, after an initial decrease in the percentage of viable cells (statistically significant for PE 250 μg/mL treatment with respect to vehicle), there was a consistent recovery in cell viability starting at 13 days of exposure (Figure 5a). We followed the cell cultures through optical microscopy during chronic treatment, and we observed that, at the same timepoint, perinuclear vesicles disappeared, as the degree of N/MPL uptake seemed to gradually decrease (Figure 5b). Since VK2 E6/E7 viability was fully rescued at 13 days of chronic exposure, we decided not to investigate mRNA levels for apoptosis and cell cycle markers. In addition, the expression of proliferation and differentiation genes was assessed through RT-qPCR (Figure 5c). As for *MYC*, we highlighted significant fluctuations in mRNA levels, especially for PE 250 μg/mL exposed cells with respect to control, with an increase at 3 and 7 days, and a reduction at 13 and 17 days, whilst at 21 days, *MYC* expression was restored at levels comparable to vehicle. *FGFR2 IIIB* downregulation was protracted until 17 days of treatment for PE-exposed cells compared to control, with a gradual re-establishment of mRNA physiological levels, and totally restored at 21 days. In order to monitor the activation of inflammatory response in VK2 E6/E7 cells chronically exposed to PE N/MPLs, we performed multiplex cytokine analysis on the supernatant of cells treated with low and high concentrations of PE particles for up to 21 days. As previously described, the only detectable factor was VEGF, which showed a significant increase through the experimental timepoints in PE 250 μg/mL-treated cells compared to vehicle, but returned to physiological levels starting from 17 days of exposure (Figure 5d). As concerns cells treated with PE 25 μg/mL, they showed fluctuating VEGF levels throughout the experiment that returned comparable to the expression in control cells at 21 days. However, concerning the expression of the epigenetic enzymes described in the previous paragraph, via RT-qPCR, we highlighted significant fluctuations throughout the days of treatment both for low and high PE particle concentrations (Figure 5e). In detail, we observed a sustained dysregulation overtime for *TET3* expression, with a tendency to upregulation in VK2 E6/E7 exposed to both PE particle concentrations, even at 21 days of exposure. With regards to *DNMT3B*, its expression levels were significantly downregulated in VK2 E6/E7 exposed to low and high concentrations of PE N/MPLs from day 7 to day 17 with respect to control, while at day 21, there was a significant increase of *DNMT3B* mRNA levels for PE 25 μg/mL treatment. However, in cells exposed for 21 days to PE 250 μg/mL, the downregulation of *DNMT3B* expression was still consistent. *DNMT3A* mRNA levels were also impacted by PE N/MPL treatment overtime, with a pattern comparable to that of *DNMT3B*. As for *DNMT1*, *TET1*, and *TET2* mRNA levels, the influence of PE N/MPL treatment was evident, but moderate. Considering the risk of cell transformation posed by the alteration of DNA methylation regulatory enzymes, we decided to assess the efficiency of colony formation of VK2 E6/E7 chronically exposed to low concentrations of PE N/MPLs for one month and a half. As shown by crystal violet staining and verified through the calculation of crystal violet absorbance, no substantial differences were highlighted between N/MPL- and vehicle-treated cells (Figure 5f).

## 3. Discussion

Nowadays, the extensive use of plastic in every field of life makes it quite impossible not to come into contact with this material. In addition to the common plastic items employed in everyday life as bottles, food packaging, clothing, and cosmetics, personal care products such as tampons and period pads also contain plastic material [11]. These objects may release N/MPL particles when using them, which, combined with the ubiquitous presence of N/MPLs in the environment, such as in water or food [12], might determine the enrichment of N/MPLs in the body, hence representing a potential health threat for humans. Among the different entry routes, humans are exposed to N/MPLs through the epidermis, which is the most external tissue of the body, but few works have addressed the consequences of N/MPL exposure on mammalian keratinocytes [9,17]. Moreover, most of the studies performed on epithelial cells tested only PS nano/microparticles with a particle size that was relatively large with respect to cell dimensions [43], whilst it is widely accepted that as particle size decreases, interaction with tissue and individual cells increases. Hence, particles in the nanometre size range are more likely to translocate through the skin and enter the keratinocytes [12]. The vaginal mucosa is a multi-layered stratified squamous epithelium that structurally resembles the epidermis in many respects [44]. It is constituted by keratinocytes and might be more affected than skin by N/MPL exposure, since it shows a stratum corneum that is less thick and more permeable, and it forms folds (transverse ridges or rugae), providing the vagina with increased surface area for extension and stretching. The work performed by Pantoja Munoz et al. [11] highlighted that some period products released fibres under conditions that mimic normal use. The synthetic fibres made of plastic polymers underwent fragmentation during in vitro tests, suggesting the possible release of billions of NPs per tampon in the vaginal canal. At present, the implications for women’s health are unknown. Therefore, the aim of the present study was to determine the biological effects of a mixture of PE nano/microparticles on human vaginal keratinocytes after both single-dose (acute), discontinuous, and repetitive (chronic, long-term) exposure. The treatment timepoints were intended to mimic common period product usage, particularly tampons, which are in closer contact with vaginal mucosa with respect to period pads. Indeed, they can be used sporadically, or on a regular basis by menstruating women.

By employing a human cell line of normal vaginal keratinocytes, we assessed cell vitality after acute PE N/MPL exposure and highlighted a decrease in the number of viable cells caused by the induction of apoptosis in a concentration-dependent fashion. In this regard, it has been previously demonstrated that apoptosis and mitochondrial dysfunction are consequences of PS nanoparticle exposure [45,46]. Other processes related to a reduced rate of proliferation, such as cell cycle arrest, senescence, or autophagy, were not influenced by the treatment with PE N/MPLs, but we observed a significant upregulation of *CDKN1A* (p21) mRNA level in vaginal keratinocytes treated with the highest concentration of PE particles. Furthermore, we noticed the constant formation of vesicular structures around the nucleus of vaginal keratinocytes treated with a single dose of PE N/MPLs at the highest concentration, which at TEM observation, were found to be intracellular aggregates of nanosized PE particles entrapped in endosomal-like structures. Moreover, the TEM acquisitions revealed the accumulation of PE N/MPLs between cell junctions at the lowest treatment concentration. These findings suggest that vaginal keratinocytes acutely exposed to PE N/MPLs react in a different way depending on the amount of N/MPLs that surround them; at low PE concentration, cells were able to contrast the entry of the particles, whilst at high PE concentration, cellular uptake of the nanoparticles was unavoidable. This process could be fostered by the formation of a “protein corona,” which is likely to occur in the normal vagina microenvironment. Indeed, we observed an amplification of the effects of PE N/MPL uptake on vaginal keratinocytes after acute exposure to keratin-coated PE N/MPLs, with the number of apoptotic-like cells increasing in a concentration-dependent manner. Indeed, previous in vitro experiments demonstrated that protein corona formation enables PS nanoparticles to translocate at greater rates [47], and increases the occurrence of cell interactions and toxicity [48]. With regards to N/MPL cellular uptake, previous studies showed that nanoparticles enter the cells mainly via macropinocytosis, along with clathrin- and caveolae-mediated endocytosis, which produces vesicles that firstly fuse with early endosomes, which in turn join the lysosomes [49,50,51]. However, lysosomal enzymes cannot degrade plastic polymers. As demonstrated by Zhou et al. [52], nanoparticles largely agglomerate at lysosomes upon cellular internalisation, may destabilise lysosomal membranes, and impede nanoparticles clearance from the cells, thus hindering the autophagic process and eventually triggering cell death. Hence, we speculated that the decrease in the number of viable vaginal cells caused by apoptosis might be attributable to alterations in the endosomal–lysosomal system, which in turn determine cell stress and death.

Considering the impact on cell morphology of PE N/MPLs that we highlighted by TEM, we underlined a transient breakdown of the actin cytoskeleton in vaginal cells exposed to the highest concentration of PE particles. Indeed, the actin cytoskeleton is responsible for the structural stability of cells by maintaining and adapting the cellular shape, and mediating cell–cell and cell–matrix adhesion [53]. Further, we observed β-catenin accumulation at the cell junction upon low PE particle concentration exposure, while it moved towards the nucleus at high PE N/MPL concentrations. The actin-associated protein β-catenin is involved in regulating cellular adhesion of the E-cadherin complex, and it also acts as an intracellular signal transducer in the Wnt signalling pathway [18]. So, β-catenin localisation might be related to the dual behaviour of vaginal keratinocytes exposed to different amounts of PE particles. Interestingly, Schmidt et al. [17] demonstrated that individual components of the actin cytoskeleton (e.g., stress fibres, filipodia, lamellipodia, focal adhesions) were strongly changed upon PS N/MPL treatment, which also influenced Wnt/β-catenin signalling. However, our findings suggested that whole β-catenin protein levels were diminished in PE N/MPL-treated cells at both concentrations compared to vehicle, as it was E-Cadherin expression, probably reflecting the disassembly of adherens junctions and loss of cell adhesion [54]. Similarly, we observed the dual behaviour of N/MPL-exposed keratinocytes in regard to total cytokeratin protein expression; vaginal cells exposed to low N/MPLs concentrations expressed higher levels of cytokeratins with respect to vehicle and PE 250 μg/mL-treated cells, in accordance with TEM observations. It can be speculated that cells that encounter low amounts of N/MPLs try to protect themselves from mechanical stress by reinforcing the adherens junctions (endangered by N/MPLs) through β-catenin accumulation, and by increasing the levels of cytokeratin in the cytoplasm. On the other hand, cells exposed to high concentrations of N/MPLs, no longer able to contrast particle uptake, react by activating cell stress response pathways, as previously reported by Gopinath et al. [9].

With regards to cell stress markers, we mainly observed a significant upregulation of *GPX4*, an enzyme that is activated to protect cells from lipid peroxidation [21], in vaginal keratinocytes upon PE N/MPL exposure at both concentrations. Noteworthy, it was recently demonstrated that N/MPLs can destabilise lipid membranes [46,55], and this might trigger lipid peroxidation when PE particles accumulate both in the intercellular and the intracellular compartment. We also observed the upregulation of *NRF2* expression in N/MPL-treated cells at 48 h, which also showed upregulated levels of *CDKN1A*. Indeed, p21 could be induced in response to oxidative stress and competes with Keap1 for the binding to NRF2, thus compromising NRF2 ubiquitylation and promoting the NRF2-dependent antioxidant response [56].

Contaminant elements may affect miRNA expression and exert detrimental effects on the post-transcriptional regulation of many downstream targeted genes [57]. Qu et al. identified several dysregulated miRNAs by nano-PS exposure in nematodes [58], and Barguilla et al. found a large impact of PS nanoparticles long-term exposure on the expression of miRNAs related to cell transformation processes in mouse embryonic fibroblasts [59]. In the present study, we highlighted significant dysregulation of miRNAs related to epithelial barrier function, cell stress, and inflammation in vaginal cells acutely exposed to PE N/MPLs. Noteworthy, the association between miRNA dysregulation and keratinocyte proliferation/differentiation has been previously reported for relevant skin diseases, such as atopic dermatitis and psoriasis [22]. To the best of our knowledge, the present study is the first one assessing the effects of PE N/MPLs on the expression of miRNAs in human keratinocytes.

After evaluating the effects of acute exposure to N/MPLs, we studied the fate of vaginal keratinocytes during the suspension of PE particle treatment. Apparently, cell viability and phenotype were restored back to physiology in six days, as it was for mRNA levels for genes related to apoptosis, even if these observations may be justified mostly by the disappearing of cells that ingested N/MPLs and underwent programmed cell death. *CDKN1A* mRNA level upregulation persisted even after a 144 h wash-out, suggesting the constant action of p21 on cell stress pathways. As for *FGFR2 IIIB* expression, after a significant downmodulation upon 48 h N/MPL exposure, mRNA levels returned comparable to vehicle at 144 h wash-out, this possibly indicating a transient and reversible effect on cell proliferation rate, in line with the result of cell viability assays. Concerning the expression of *MYC*, it stayed upregulated even after the wash-out of treatment. Interestingly, the activation of MYC decreases the expression of genes that encode proteins mediating cell adhesion and cytoskeletal assembly in many cell types [28], in accordance with what we observed in PE N/MPL-exposed vaginal keratinocytes. Moreover, MYC activation inhibits cell-extracellular matrix adhesion, cell spreading and cell motility in cultured keratinocytes and impairs stem cell maintenance and epidermal wound healing in vivo [60]. Regarding MUC1, its N-terminal subunit is an integral component of the cell surface glycocalyx and mucous gel and contributes to the physical protection of barrier tissues, and the MUC1-C terminal subunit has the capacity for activating stem cell functions in the repair and remodelling of the barrier and for inducing the re-establishment of homeostasis upon resolution of inflammation [61]. Hence, the downregulation of *MUC1* expression, along with the upregulation of *MYC* after wash-out, may be a persistent side effect of acute N/MPL exposure, which might influence the regenerative capability of the vaginal mucosa.

Regarding inflammation, we tested the levels of several pro-inflammatory cytokines in the supernatant of vaginal keratinocytes discontinuously exposed to PE N/MPLs and found that only VEGF was detectable and dysregulated. Interestingly, VEGF has been suggested as a potential biomarker for dermal impairment and keratinocyte damage [58]. Indeed, epidermal keratinocytes are a major source of this growth factor, and it was demonstrated that VEGF plays a crucial albeit nonessential role during epidermal wound healing, which is substantially retarded in its absence [62]. Notably, we found VEGF to be downmodulated in the supernatant of cells treated with the lowest concentration of PE particles, and this effect persisted even after 6 days of wash-out from treatment, once more suggesting that N/MPL exposure may hinder the regenerative ability of vaginal mucosa.

With regards to the dysregulated expression of DNA methyl transferases, it is worth noting that at 144 h, wash-out mRNA levels of *DNMT1*, *DNMT3A* and *DNMT3B* were more impacted compared to a 48 h treatment, with the downregulation of the formers and upregulation of the latter. In the skin, *DNMT1* is expressed in the basal cells of the epidermis and is known to decrease with differentiation. Consistent with this, the depletion of DNMT1 in human epidermal stem cells (EpSC) promotes premature, irreversible differentiation. DNMT3A and DNMT3B play important roles in regulating human EpSC homeostasis via their ability to promote enhancer activity of genes involved in self-renewal and differentiation [63]. For what concerns the expression of DNA demethylases, we noticed a general downregulation of their level in vaginal keratinocytes after discontinuous PE N/MPL exposure. It has been proposed that reduced levels of TET1 and, subsequently, 5-hydroxymethylcytosine cause the impaired self-renewal of stem cells [64]; Liu et al. demonstrated that TET2 regulated cell viability, apoptosis and the expression of inflammatory mediators in keratinocytes [65]; Ghaffarinia et al. observed diminished 5-methylcytosine and 5-hydroxymethylcytosine amounts, and decreased mRNA expression of *TET3* in psoriatic epidermis [66]. Even if little is known about the role of epigenetic enzymes in keratinocyte biology, overall, our findings suggest that the dysregulation of DNMTs and TETs levels upon N/MPL exposure might accelerate processes leading to vaginal cell ageing.

One of the most alarming aspects of the pervasive N/MPL pollution is the potential health effects prompted by their long-term exposure. The vast majority of in vitro experimental settings involved high concentrations and short exposure times to N/MPLs, not comparable to the real exposure scenario, where individuals are chronically exposed to low concentrations of N/MPLs. Up to date, very few studies investigated detrimental biological outcomes associated with long-term N/MPL exposure [59,67,68]. Although the vagina is characterised by self-cleaning properties, its epithelium shows muco-adhesion properties and might retain nanosized plastic particles [11]. Considering this, we tested the effects of 21-day PE N/MPL constant exposure on vaginal keratinocytes, both at low and high concentrations. Interestingly, we demonstrated good resilience with totally recovered cell viability and morphology, normalisation in the expression of genes related to cell proliferation and differentiation, as well as supernatant-derived VEGF levels, which were restored to physiology after 3 weeks of exposure. Notably, *MUC1* mRNA levels remained upregulated after 17 days of treatment, possibly indicating that cells adapted to the chronic particle exposure and produced a thicker glycocalyx to contrast the uptake of N/MPLs. More in general, the lower toxic effect of N/MPLs highlighted with continuous exposure suggests cellular adaptation to stress, as also demonstrated by Schmidt et al. [17]. However, the expression of the previously mentioned epigenetic enzymes showed a fluctuating pattern throughout the experimental points, and persisted altered even after 21 days of exposure, implying that the cell adaptation to N/MPLs might be only apparent. We speculated that, in the long run, these vaginal keratinocytes driven by the sustained epigenetic imbalance might undergo two different fates: accelerated cell ageing or malignant transformation. The ageing process is accompanied by a series of prominent hallmarks, including genetic and epigenetic alterations, the latter including DNA methylation modification [69]. Environmental long-term exposure to contaminants is a strong hazard for carcinogenesis, but there is a lack of information on malignant transformation risk associated with long-term exposures to N/MPLs. Barguilla et al. exposed murine cells to PS nanoparticles for 6 months, which showed an exacerbation of several hallmarks of cancer [59]. In the present study, we tested the clonogenic potential of vaginal keratinocytes exposed to low PE N/MPL concentration for up to 6 weeks, as the formation of clones is typical of transformed cells with tumour-initiating capabilities. However, in this regard, we did not observe significant differences between vehicle and PE particle-treated cells. Since tumour induction is a process consisting of multiple steps and requiring prolonged exposures, it is important to adopt long-term experimental approaches to gain deep insight into the potential carcinogenic risk of N/MPL exposure.

The present study may have some limitations. One of them is that we only employed plain spherical PE N/MPLs for our experiments, but the nanoparticles which may come in contact with vaginal mucosa in a real scenario are unevenly multi-shaped and made of several polymers and plastic additives. Indeed, future analyses should be performed by using genuine mixtures of environmentally retrieved plastic particles. Another issue is that 2D cultures do not exactly reproduce in vivo conditions, so, to mimic the human vaginal canal, we will determine the response to N/MPLs by using 3D models based on microfluidic chips [70], novel engineered systems that act as an intermediate between a well-plate system, and animal models. We will employ primary vaginal keratinocytes for the assembly of these chips, despite the difficulties of obtaining a biopsy of vaginal mucosa from healthy donors, mainly due to the discomfort that such an intervention may bring with it. By using the vagina-on-chip, we will also address the impact of PE N/MPLs on vaginal microbiota, since dysbiosis is strictly related to gynaecological diseases, such as bacterial vaginitis and vulvovaginal candidiasis, which may be associated with various other reproductive tract disorders such as infertility, preterm, and cervical cancer [71]. The ability of microfluidic systems to recapitulate in vivo-like microenvironments and responses will offer a new avenue for assessing potential risks of N/MPL exposure and delineating their biological mechanisms.

## 4. Materials and Methods

### 4.1. Nano/Microplastics and Reagents

Polyethylene nano/microplastics (PE N/MPLs) 200 nm–9900 nm size range (Cospheric LLC, Santa Barbara, CA, USA) were resuspended in 1% Tween20 (Dako, Agilent Technologies, Santa Clara, CA, USA) solution to obtain a 100 mg/mL stock, following the manufacturer’s directions. Working solutions were made by diluting the stock solution with ultrapure distilled H_2_O so that the final concentration of Tween20 in the culture media was maintained below 0.01% to minimise the surfactant-mediated cytotoxicity [72]. Stocks and working solutions were thoroughly vortexed before use. PE N/MPLs were used at 25 and 250 μg/mL in cell culture assays. After excluding that matching volumes of Tween20 alone could have effects on cells in different experimental settings, a vehicle control containing the same amount of Tween20 as that of the highest concentration of PE working solution was employed.

### 4.2. Cell Cultures and Treatments

VK2 E6/E7 cell line (ATCC CRL-2616) was established from the normal vaginal mucosal tissue taken from a premenopausal woman undergoing anterior–posterior vaginal repair surgery. We performed our experiments on a single type of cell line, since, to the best of our knowledge, VK2 E6/E7 is the only healthy vaginal model available on the market. VK2 E6/E7 cells were grown in a Keratinocyte-Serum Free Medium (K-SFM; GIBCO, Carlsbad, CA, USA) with 0.1 ng/mL human recombinant epidermal growth factor (EGF), 0.05 mg/mL bovine pituitary extract, additional calcium chloride 44.1 mg/L (final concentration 0.4 mM), and 1% penicillin/streptomycin (Aurogene, Rome, Italy). Cells were maintained at 37 °C in a saturated humidifying 5% CO_2_ incubator. Cells were treated with vehicle (Veh; 0.0025% Tween20) and 25 and 250 µg/mL PE N/MPLs for 48, 72, and 120 h (acute exposure) for 21 days (chronic exposure), and with vehicle and 25 µg/mL PE N/MPLs for 1.5 months (low concentration, extended time chronic exposure). Cells were always sub-cultured at 80% confluence since keratinocytes spontaneously start differentiating at the higher confluence. Optical microscopy pictures of N/MPLs-treated cells were taken at 40× magnification by using EVOS XL Core Imaging System (Thermo Fisher Scientific, Waltham, MA, USA).

### 4.3. Cell Viability Assays

Trypan blue dye exclusion assay (Sigma-Aldrich, St. Louis, MO, USA) was performed to assess cell viability at 48, 72, 120, and 144 h, and 3, 7, 13, 17, and 21 days from treatment. The samples were labelled with Trypan blue in a 1:1 ratio and counted by using the Countess II Automated Cell Counter (Thermo Fisher, Waltham, MA, USA). To assess cell proliferation in wash-out experiments, cells were seeded onto 96-well plates at a density of 3 × 10^3^ cells/well, treated with vehicle, PE 25 and 250 µg/mL for 48 h, and then the medium was replaced by fresh N/MPLs-free medium for 144 h. At the end of each time point, cells were incubated with 0.5% MTT (3-(4,5-dimethylthiazol-2-yl)-2,5-diphenyltetrazolium bromide) (Sigma, St. Louis, MO, USA) for 3 h at 37 °C. The supernatant was then discarded, the MTT was dissolved with 100 μL of DMSO, and the absorbance was read at OD = 550 nm with an ELISA Microplate Reader (Bio-Rad, Hercules, CA, USA). Cell viability in PE-treated cells was calculated in comparison to control samples (vehicle), arbitrarily set to 100%, having six determinations per assay for each experimental condition.

### 4.4. Cell Cycle Analysis

VK2 E6/E7 cells exposed to vehicle or PE (25–250 μg/mL) for 48, 72, and 120 h were collected and fixed overnight at 4 °C in 70% ice-cold ethanol. After fixation, cell pellets were washed twice with ice-cold phosphate-buffered saline solution (PBS) and treated with RNase A for 15′ at 37 °C. Propidium iodide (PI, Sigma-Aldrich; Merck KGaA, Darmstadt, Germany) was added to each sample and DNA content was determined by collecting 10,000 events by using a CytoFLEX Flow Cytometer (Beckman Coulter Life Sciences, Indianapolis, IN, USA). Acquisition analysis was performed with CytExpert software 2.3 (Beckman Coulter Life Sciences).

### 4.5. Apoptosis Analysis

Apoptosis occurrence in vehicle and PE (25–250 μg/mL)-treated cells (72 h) was analysed by flow cytometry using the PE-Annexin V Apoptosis Detection Kit I (BD Pharmingen™, BD Bioscience Franklin Lakes, NJ, USA), as previously described [73]. Control cells were treated with 0.0025% Tween20. A quantity of 2 × 10^5^ cells for each experimental condition was processed for PE Annexin V and 7-AAD staining; then samples were acquired with CytoFLEX Flow Cytometer, and acquisition analysis was performed with CytExpert software to distinguish and estimate the percentage of living, dead, early, and late apoptotic cells.

### 4.6. Quantitative Real-Time PCR (RT-qPCR)

Total RNA from vaginal cells was extracted by using TRIzol reagent (Invitrogen, Carlsbad, CA, USA) and quantified through a NanoDropTM 2000c spectrophotometer (Thermo Fisher Scientific, Waltham, MA, USA). RNA (1–2 µg) was reverse transcribed with the High-Capacity RNA to cDNA Kit (Applied Biosystems by Thermo Fisher Scientific), according to the manufacturers’ instructions. Specific gene expression was evaluated by RT-qPCR experiments with TaqMan Gene Expression Assays (*TP53*, Hs01034249_m1; *CDNK1A*, Hs00355782; *CDKN2A*, Hs00923894_m1; *BAX*, Hs00180269_m1; *BCL-2*, Hs04986394_s1; *MYC*, Hs00153408_m1; *MUC1*, Hs00159357_m1; *FGFR2IIIB*, custom assay [74]; *TET1*, Hs04189344_g1; *TET2*, Hs00325999_m1; *TET3*, Hs00896441_m1; *DNMT1*, Hs00154749_m1; *DNMT3A*, Hs01027166_m1; and *DNMT3B*, Hs00171876_m1—Applied Biosystems by Thermo Fisher Scientific) and TaqMan miRNAs Expression Assays (hsa-miR-29a-3p, ID:002112; hsa-miR-34a-5p, ID:00426; hsa-miR-124-5p, ID:002197; hsa-miR-125b-5p, ID:000449; hsa-miR-145-5p, ID:002278; hsa-miR-200c-3p, ID:002300; and hsa-miR-378a-3p, ID:002243—Applied Biosystems by Thermo Fisher Scientific). The housekeeping Cyclophilin-A mRNA (*PPIA*, Hs_04194521_s1—Applied Biosystems by Thermo Fisher Scientific) or U6 snRNA (ID:001973—Applied Biosystems by Thermo Fisher Scientific), whose expression was not affected by PE N/MPL exposure, were used as endogenous controls. The expression of genes involved in redox homeostasis was assessed through SYBR green RT-qPCR analysis, by employing the following primers: *NRF2* (Fw GAGAGCCCAGTCTTCATTGC; Rev TTGGCTTCTGGACTTGGAAC); *KEAP1* (Fw TGGCCAAGCAAGAGGAGTTC; Rev GGCTGATGAGGGTCACCAGTT); *SOD2* (Fw TGCACTGAAGTTCAATGGTGG; Rev CTTTCCAGCAACTCCCCTTTG); *CAT* (Fw TAAGACTGACCAGGGCATC; Rev CAAACCTTGGTGAGATCGAA); *GPX4* (Fw AGACCGAAGTAAACTACACTCAGC; Rev CGGCGAACTCTTTGATCTCT); and *GSTM1* (Fw AGAGGAGAAGATTCGTGTGG; Rev TGTTTCCTGCAAACCATGGC). Samples were run on AB Step One Real-time PCR System (Applied Biosystems by Thermo Fisher Scientific), and results were analysed as previously described [75].

### 4.7. Senescence Analysis by β-Galactosidase Staining

Cellular senescence was visualised and quantified by measuring the activity of senescence-associated β-galactosidase with a Senescence β-Galactosidase Staining Kit (Cell Signalling, Danvers, MA, USA), according to the manufacturer’s instructions. Briefly, cells were exposed to vehicle or PE N/MPLs (25–250 µg/mL) for 72 h and to H_2_O_2_ as a positive control; then, cells were trypsinised, and 5 × 10^5^ cells were seeded in a 6-well plate without treatment. After 24 h settlement, cells were washed with PBS and fixed with the Fixative Solution for 15 min at room temperature (RT), followed by staining with the β-galactosidase Staining Solution, as previously described [76]. Cells were incubated for 24 h at 37 °C in a dry incubator, and then pictures were taken at 40× magnification by using EVOS XL Core Imaging System (Thermo Fisher Scientific).

### 4.8. Immunofluorescence

VK2 E6/E7 cells were plated in 24-well plates with 2% gelatin-coated glasses (6 × 10^4^ cells/well). After 24 h of settlement, cells were treated with vehicle or PE N/MPLs (25 and 250 µg/mL) for 72 h. Immunofluorescence of cells fixed in 4% paraformaldehyde was performed as previously described by Ceccarelli et al. [77]. Cells were incubated for 1 h at RT with anti-Lamin B and anti-β-Catenin primary antibodies (Santa Cruz Biotechnology, Dallas, TX, USA), followed by a specific Alexa Fluor™ 488-conjugated secondary antibody (Thermo Fisher Scientific) for 30 min. Actin filaments were labelled with DyLight™ 554 Phalloidin (Cell Signalling Technology, Danvers, MA, USA) for 1 h at RT. Cell nuclei were stained with 1 μg/mL 4’, 6-diamido-2phenylindole dihydrochloride (DAPI, Sigma-Aldrich). Images were acquired with a fluorescence microscope (Olympus BX53, Center Valley, PA, USA) using a 40× magnification.

### 4.9. Western Blot Analysis

VK2 E6/E7 cells treated with vehicle or PE N/MPLs for 48 or 72 h were lysed in RIPA buffer. Total protein extracts were separated on 10% or 15% SDS-PAGE and underwent Western blot analysis as previously described [78]. Membranes were incubated overnight at 4 °C or 1 h at RT with the following primary antibodies: anti-p62 (BD Transduction Laboratories, Franklin Lakes, NJ, USA), anti-LC3 II/I (Sigma Aldrich), anti-Pan Keratin C11 (Cell Signalling Technology), anti-E-cadherin, and anti-β-Catenin (all of them by Santa Cruz Biotechnology). Since N/MPLs treatment might affect cytoskeleton organisation, we initially tested both β-Actin and glyceraldheyde-3-phosphate dehydrogenase (GAPDH) protein levels as endogenous controls. We verified that actin expression was not affected by the treatment, and finally employed anti-β-Actin (Santa Cruz Biotechnology) as the loading control for our experiments. Images were acquired by ChemiDoc XRS+ (Bio-Rad, Hercules, CA, USA). Densitometry was performed with ImageJ 1.5 software [79], and band intensities were shown as the fold changed to the corresponding control.

### 4.10. Electron Microscopy

To assess the possible PE N/MPL uptake by vaginal cells and highlight the presence of intracellular PE N/MPLs, we performed TEM experiments. VK2 E6/E7 cells, cultured and treated as described above, were fixed in 2% glutaraldehyde (Electron Microscopy Sciences, Hatfield, PA, USA) in PBS for 24 h at 4 °C. After collection, cells were washed three times in PBS, post-fixed for 2 h in 1% OsO_4_ (Electron Microscopy Sciences), dehydrated in graded acetones and embedded in Epon-812 (Electron Microscopy Sciences) for 48 h at 60 °C. Ultrathin sections (60 nm) were cut with a Reichert ultramicrotome (Leica Microsystems, Wetzlar, Germany), mounted on copper grids, counterstained with uranyl-acetate replacement stain and lead citrate (Electron Microscopy Sciences) and finally examined with a Philips CM10 (TEM) and/or a Fei-Philips Morgagni 268D transmission electron microscope (FEI, Eindhoven, The Netherlands). Furthermore, to mimic the formation of a protein corona [9], we employed PE N/MPLs coated with keratin from human epidermis (Sigma-Aldrich), by incubation for 24 h at 4 °C prior to the treatment.

### 4.11. Multiplex Cytokine Analyses of Cell Culture Supernatants

The effects of PE N/MPL exposure on VK2 E6/E7 inflammatory response were evaluated in cell culture supernatants by testing 10 cytokines. We used specifically customised human kits (R&D System, Biotechne Minneapolis, MN, USA) with a Luminex MAGPIX detection system and followed the manufacturer’s instructions. In detail, we evaluated: TSLP, CXCL1, CXCL10, TNF-α, VEGF, and IL-1α, IL-1β, IL-6, IL-12, and IL-33. The levels of cytokines (pg/mL) were estimated using a 5-parameter polynomial curve (Bio-Plex Manager 6.2 software, Bio-Rad, California, USA).

### 4.12. Colony Formation Assay

VK2 E6/E7 cells were treated for six weeks with vehicle or PE 25 μg/mL, and then 2 × 10^3^ cells/well were plated in a 6-well plate. The medium was replaced every 3 days, and after 12 days, colonies were stained with 0.1% crystal violet for 15 min at RT. Colonies were photographed, and then crystal violet was solubilised in 30% acetic acid in water for 15 min at RT; absorbance was measured by using the Biochrom Libra S22 UV/VIS spectrophotometer (Biochrom, Berlin, Germany) at a wavelength of 595 nm; 30% acetic acid in water was used for the blank [80].

### 4.13. Statistics

For each experiment, at least three replicates were performed, and all results are expressed as the mean ± SD. Statistical significance was calculated with GraphPad Prism 8.0.1 (GraphPad Software Inc., San Diego, CA, USA) using Student’s *t*-test. *p*-values less than 0.05 were considered statistically significant.

## 5. Conclusions

Herein, we investigated the effects of PE N/MPLs on vaginal keratinocytes following different exposure modalities. We highlighted substantial N/MPL uptake by vaginal cells after acute exposure, which significantly affected cell vitality through the activation of apoptosis and influenced cytoskeleton organisation and cell stress pathways. When the exposure to PE N/MPLs was discontinued or became chronic, cells were able to recover from the detrimental effects on viability and proliferation gene expression in a few days, suggesting the induction of adaptive processes. However, in all cases, PE N/MPL exposure determined the sustained alteration of epigenetic enzymes expression profile, which might be at the basis of accelerated cell ageing or increased risk of neoplastic conditions. With our work, we contributed to a better understanding of N/MPL-induced toxicity and to the unveiling of possible health risks related to N/MPL exposure, with the purpose of finding the best strategies to avoid the use of plastic materials in hygiene products currently used by women.

## Data Availability

The data that support the findings of this study are available from the corresponding author upon reasonable request.

## Supplementary Materials

The following supporting information can be downloaded at: https://www.mdpi.com/article/10.3390/ijms241411379/s1.
