# Supplementary material for: Assessing the Impact of Polyethylene Nano/Microplastic Exposure on Human Vaginal Keratinocytes
Source: Int J Mol Sci. 2023 Jul 12;24(14):11379. doi: 10.3390/ijms241411379 (PMC10380279; doi:10.3390/ijms241411379)

SUPPLEMENTARY MATERIALS

Figure S1. TEM images with marked endo-vesicular PE N/MPL size.

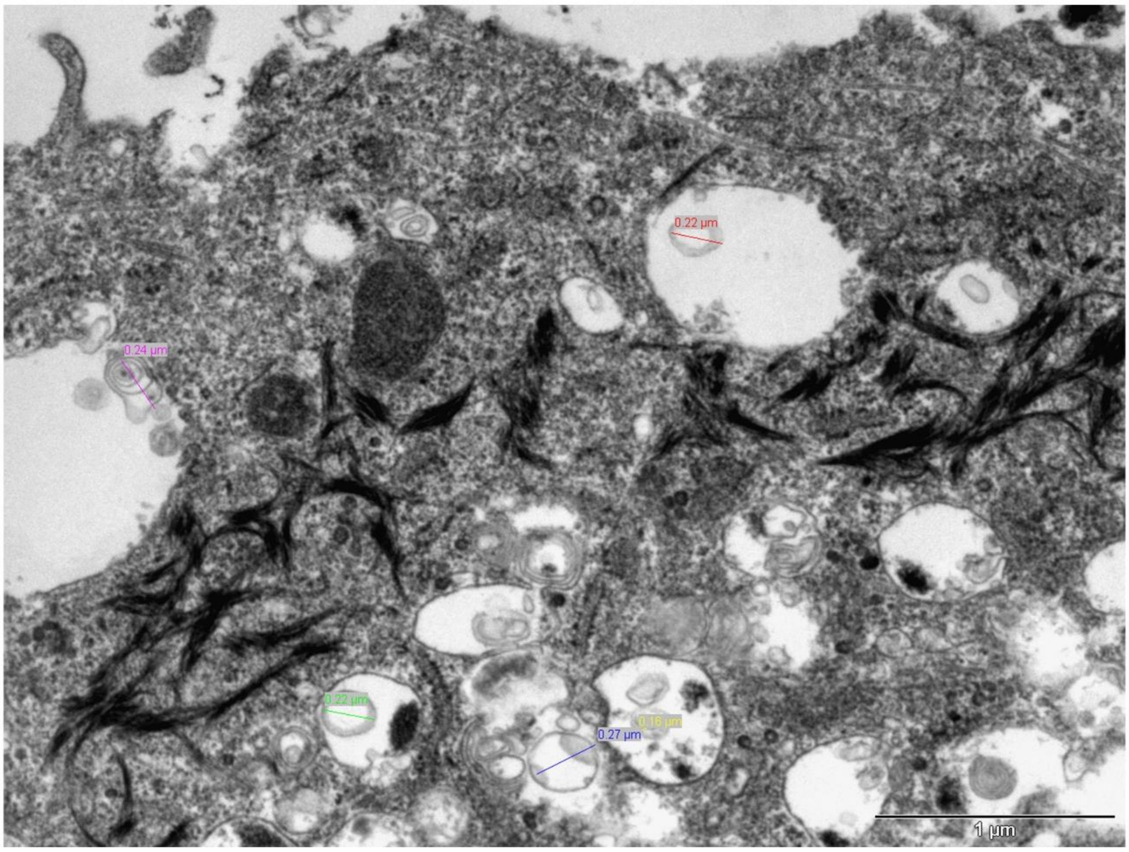

Table S1. Endovesicular PE N/MPL mean diameter calculation.

**Pic 1**	**Pic 2**	**Pic 3**	**Pic 4**	**Pic 5**
Distance	Distance	Distance	Distance	Distance
µm	µm	µm	µm	µm
0.18	0.12	0.12	0.22	0.22
0.15	0.22	0.13	0.22	0.25
0.26	0.19	0.15	0.16	0.15
0.26	0.17	0.13	0.27	0.17
0.11	0.16	0.21	0.24	0.14
0.14	0.17	0.15		0.22
0.13	0.24	0.30		
0.15		0.17		
0.17				
**Single Pic Mean**				
0.17	0.18	0.17	0.22	0.19
**Tot Mean**				

Distance				
μm	0.18			
nm	180			

Figure S2: Uncropped Western Blot images. (a) WB in Figure 1h. **(b)** WB in Figure 3c.
The protein standard used in Western Blot analyses was Precision Plus Dual Color BIO-RAD (#1610374)

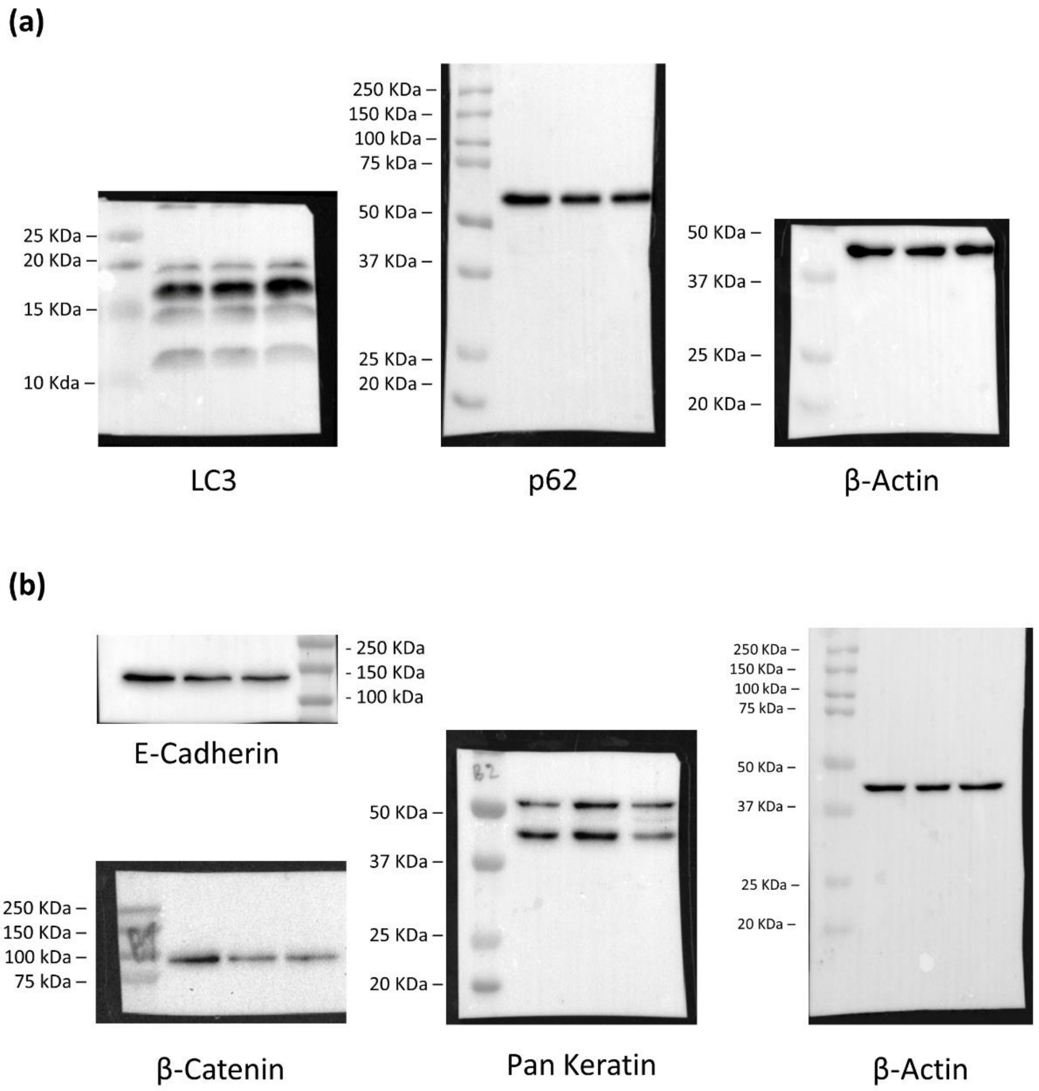

Supplement: Supplementary file 1 [file ijms-24-11379-s001.zip › ijms-2398454-supplementary.pdf]
